# Effects of Rex-bypass shunt on the cavernous transformation of the portal vein in children: evaluation by the color Doppler ultrasonography

**DOI:** 10.1186/s13244-019-0815-6

**Published:** 2020-01-03

**Authors:** Zhengmin Ruan, Mei Wu, Chunchun Shao, Yuan Zhang, Caikun Zhang, Feixue Zhang, Bin Zhao

**Affiliations:** 1 Department of Ultrasound, The Second Hospital of Shandong University, No 247, Beiyuan Street, Ji’nan, 250033 China; 2 Center for Evidence-Based Medicine, Institute of Medical Sciences, The Second Hospital of Shandong University, Ji’nan, China; 3 Department of Western Medicine, Shandong College of Traditional Chinese Medicine, Yantai, China; 4 Shandong Medical Imaging Research Institute, Jinan, China

**Keywords:** Cavernous transformation of portal vein, Rex-bypass shunt, Color Doppler ultrasonography

## Abstract

**Background:**

The study was to investigate the role of color Doppler ultrasonography in the evaluation of the effect of Rex-bypass shunt on the cavernous transformation of the portal vein (CTPV) in children.

**Methods:**

Fifty children with symptomatic extrahepatic portal hypertension who received Rex-bypass shunt were retrospectively reviewed, and they were diagnosed with CTPV by ultrasonography. The clinical characteristics were analyzed before and after operation.

**Results:**

Forty-five patients received color Doppler ultrasonography at 6 months after surgery, and good patency in the bypass vessels was displayed. The platelet count significantly increased (*P* < 0.001) and the esophagogastric varices were improved significantly (*p* < 0.001). The patency of bypass vessels on color Doppler ultrasonography was consistent with the changes in the platelet count and the degree of esophagogastric varices on gastroscopy before and after operation. The diameter of bypass vessels at 6 months was slightly larger than that at 7 days after operation, and there was no significant difference in blood flow velocity between two time points (*P* = 0.507).

**Conclusions:**

Color Doppler ultrasonography can display the patency, diameter, and flow velocity of bypass vessels. It plays an important role in evaluating the effect of Rex-bypass shunt on the CTPV.

## Key points

- Color Doppler ultrasound can clearly display the patency, diameter, and flow velocity of bypass vessels.
- Color Doppler ultrasound plays an important role in evaluating the effects of Rex-bypass shunt.
- Color Doppler ultrasound is a preferred tool for the examination after Rex-bypass shunt.

## Background

Cavernous transformation of the portal vein (CTPV) is the most common cause of portal hypertension in children. The prehepatic portal hypertension in CTPV children is caused by complete or partial portal vein obstruction. Patients with CTPV are prone to a series of severe complications including recurrent upper gastrointestinal hemorrhage and hypersplenism. About 10% of CTPV children will die of recurrent upper gastrointestinal bleeding [1]. At present, surgical treatment of CTPV is relatively difficult because the vessels have irregular courses and are susceptible to bleeding [2]. As compared to the traditional treatments (portosystemic shunt, paraesophagogastric devascularization, variceal banding ligation, splenorenal shunt, and sclerotherapy), Rex-bypass shunt is a relatively new and effective surgical intervention for the CTPV without additional liver lesions [3]. It is currently considered as a gold-standard strategy by some surgeons [4, 5]. By creating a bypass between the superior mesenteric vein and the left portal system, the splanchnic venous blood circulation should be restored [6, 7].

Ultrasonography provides a reliable and useful tool for the diagnosis and evaluation of CTPV [8]. Color Doppler ultrasonography can display the specific location of cavernous transformation [9, 10]. The clinical manifestations and treatment procedures of CTPV vary, depending on the location of cavernous transformation [11]. Previous studies have focused on the role of color Doppler ultrasonography in the diagnosis of CTPV [8, 9, 12], but little is known about the role of color Doppler ultrasonography in the evaluation of CTPV after surgical treatment, especially after Rex-bypass shunt. In this study, we retrospectively analyzed the ultrasonographic data of 50 children who underwent Rex-bypass shunt for CTPV, aiming to evaluate the value of color Doppler ultrasonography in the assessment of the effect of Rex-bypass shunt on the CTPV in children.

## Methods

### Subjects

Fifty CTPV children with symptomatic extrahepatic portal hypertension received Rex-bypass shunt in the Second Hospital of Shandong University between January 2010 and November 2017 and were included in this study. They were diagnosed with CTPV by ultrasonography. In these patients, the left intrahepatic portal vein was patent on portography before surgery. The bypass vessels included 10 internal jugular veins, 20 splenic veins, 6 great saphenous veins, 6 gastric coronal veins, 5 umbilical veins, 2 right gastroepiploic veins, and 1 external jugular vein. The mean age was 7.17 ± 3.01 years (range 3–13 years). There were 31 boys and 19 girls. During the surgery, children younger than 4 years were sedated in order to reduce the influence of breathing and avoid movement. All the children had a history of upper gastrointestinal bleeding, hypersplenism, melena, or some other symptoms. The clinical characteristics including color Doppler ultrasonographic data, laboratory findings, and endoscopic findings were collected before and after surgery and analyzed. These children were followed-up at 7 days and 6 months after operation because some studies have shown that the incidence of postoperative rebleeding in children within 6 months postoperatively was higher than that at 6–12 months or 12 months after surgery [13].

### Methods

GE LOGIQ 9 and E9 ultrasound system and convex array probe (C1-5, 4 MHz) were applied for color Doppler ultrasonography. The ultrasound examination was performed by the same experienced clinician, aiming to reduce the interobserver variation. Ultrasonographic data (including the patency, diameter, and flow velocity of bypass vessels) were collected before and after surgery and analyzed. Moreover, the platelet count and endoscopic findings and degree of esophagogastric varices were also reviewed. According to the patency of bypass vessels, children were divided into two groups: Group A, the patent bypass vessels, and Group B, the blocked bypass vessels.

### Color Doppler ultrasonography of the bypass vessels

Color Doppler ultrasonography allows the measurement of blood velocity of vessels. In this study, the angles between the long axis of vessels and the Doppler beams were < 60° in the measurement of velocity in order to achieve a higher accuracy. The center of the bypass vessel was sampled. The diameter of the bypass vessel was measured on the longitudinal section.

### Statistical analysis

Statistical analysis was performed using SPSS Statistics version 24.0. The normal distribution of continuous variables was evaluated by the Kolmogorov-Smirnov test. Student’s *t* test or nonparametric test was used to test the differences between Group A and Group B, depending on the results of normal distribution test. Chi-square test or Fisher’s test was used for the comparisons of categorical variables. Pearson and Kendall correlation were used to analyze the correlation. A value of *p* < 0.05 was considered statistically significant.

## Results

### Patency of the bypass vessels

The patency of the bypass vein after Rex-bypass shunt is an important indicator of prognosis. Two patients showed stellate blood flow signals in the bypass vessels on the color Doppler ultrasonography at 7 days after surgery, suggesting poor patency of the bypass vessels. After clinical anticoagulation treatment for 6 months, rich blood flow signals were observed in the bypass vessels in one of the above two patients. Color Doppler ultrasonography showed good patency of the bypass vessels and red blood flow signals, suggesting the blood flow into the liver, while thrombosis was observed in the bypass vessel in the other patient because blood flow signals were not found. It showed the bypass vessels were still blocked. Both patients underwent computed tomography venography (CTV), and the results of CTV were consistent with those of ultrasonography.

**Fig. 1:**
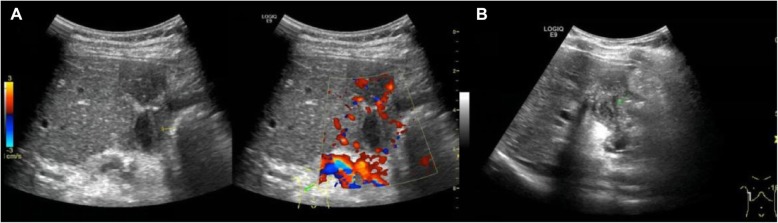
Color Doppler ultrasonography of an 8-year-old girl after Rex-bypass operation for CTPV. No blood flow signal was found in the bypass vessels, and thrombosis was observed. **a** 7 days after operation. **b** 6 months after operation

**Fig. 2:**
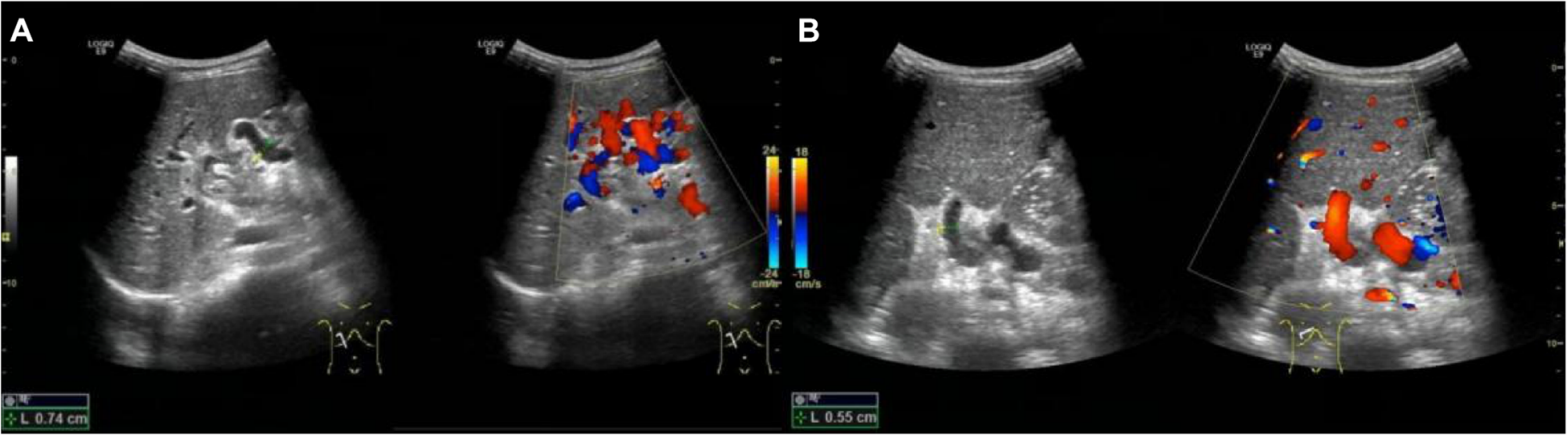
Rich blood flow signals and good patency in the bypass vessels on the color Doppler ultrasonography at 7 days after Rex-bypass operation for CTPV. **a** 7-year-old boy. **b** 6-year-old boy

In four patients, thrombosis (no blood flow signals) was identified in the bypass vessels at 7 days and 6 months after surgery (Fig. 1), suggesting the operation was a failure. These results were further confirmed by CTV. In the remaining 44 patients, blood flow was favorable and the bypass vessels had good patency at 7 days and 6 months after operation (Fig. 2). Only 20 patients underwent CTV, which also showed the patency of the bypass vessels although the results on CTV were not satisfactory.

Preoperative hepatic biopsy revealed mild nodular cirrhosis in 12 patients, chronic active hepatitis in 13, and signs of both lesions in 3. Among 50 patients, preoperative hepatic biopsy showed normal in 22 patients. The five patients with thrombosis mentioned above were diagnosed with chronic active hepatitis or mild nodular cirrhosis and chronic active hepatitis.

In summary, 45 patients received color Doppler ultrasonography at 6 months after surgery, and good patency was noted in the bypass vessels (Group A). The ultrasonographic findings were graded as 0, 1, and 2 which represent rich, stellate, and none blood flow signals in the bypass vessels, respectively, on the color Doppler ultrasonography. The bypass vessels included 3 umbilical veins, 1 internal jugular vein, and 1 great saphenous vein in the 5 patients of Group B.

### Patency of the bypass vessels based on laboratory and gastroscopic findings of esophagogastric varices

Within 6 months after operation, 12 patients in Group A developed hematemesis, black stool, and/or hypersplenism, 8 of whom underwent gastroscopic ligation of esophageal varices. All the patients in Group B developed hematemesis and blackstool and/or hypersplenism, four of whom received splenorenal shunt operation. One patient in Group B underwent gastroscopic ligation of esophageal varices.

Platelet count is an important parameter for hypersplenism. The platelet count increased slightly in three patients and decreased slightly in two patients in Group B. The changes of platelet count after Rex-bypass shunt in Group A and Group B are shown in Table 1. The relationship between the changes of platelet count and the patency of bypass vessels was analyzed in Group A and Group B. Results showed the platelet count significantly increased at 6 months after operation in the patients of Group A (good patency of bypass vessels) (*p* < 0.001). The platelet count remained unchanged after operation in Group B, but the number of patients was small. Postoperative platelet count in Group A was significantly higher than in Group B (*P* = 0.002).

**Table 1:** Platelet count before and at 6 months after Rex-bypass shunt operation in Group A and Group B

	PLT (10^9^/L)		*Z*	*P*
	Group A	Group B		
Pre-operation	79.00 (62.50, 92.50)	70.00 (53.50, 104.50)	0.243	0.808
Post-operation	295.00 (196.50, 363.00)	69.00 (59.00, 85.00)	3.169	0.002
*Z*	10.633			
*P*	< 0.001			

**Fig. 3:**
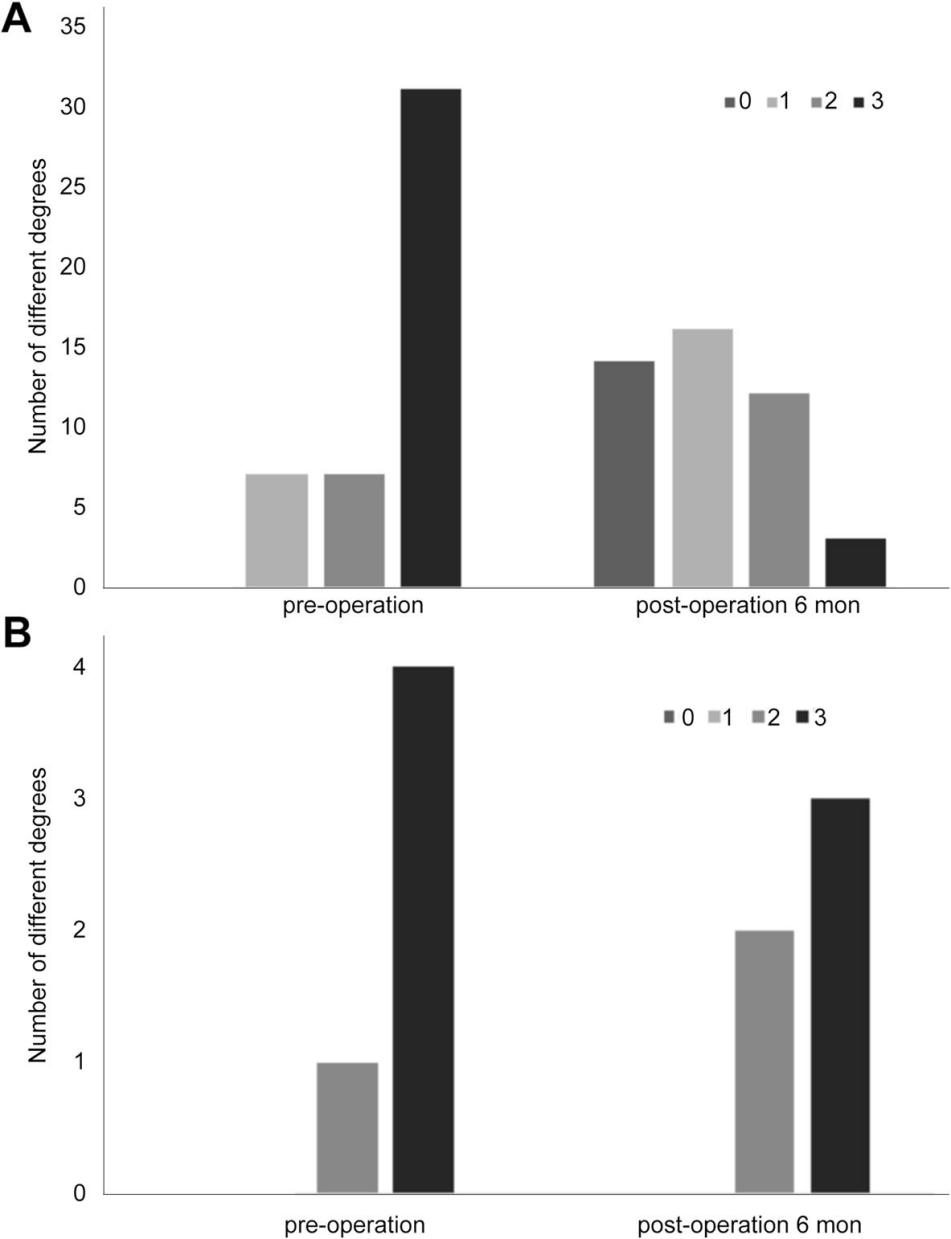
Degrees of esophagogastric varices in Group A and Group B before and at 6 months after Rex-bypass shunt on gastroscopy. **a** Group A. **b** Group B

The patients with different degrees of esophagogastric varices in Group A and Group B are shown in Figs. 3a and b. The esophagogastric varices were graded 0, 1, 2, and 3 to reflect none, mild, moderate, and severe esophagogastric varices, respectively. The esophagogastric varices in Group A were improved significantly at 6 months after operation (Table 2, *p* < 0.001).

**Table 2:** Degrees of esophagogastric varices before and at 6 months after Rex-bypass shunt by gastroscopy

	Alleviative	Unchanged	Aggravated	*χ*^2^	*P*
Group A (*n* = 45, %)	40 (88.9)	2 (4.4)	3 (6.7)	14.738	< 0.001
Group B (*n* = 5, %)	1 (20.0)	4 (80.0)	0 (0.0)		

These results suggest that the patency of bypass vessels on color Doppler ultrasonography is consistent with the change in the platelet count and the degree of esophagogastric varices on gastroscopy.

### Diameter and flow velocity of bypass vessels after operation

The flow velocity and diameter of bypass vessels measured by color Doppler ultrasonography are useful for the evaluation of the patency of bypass vessels. The diameter of the bypass vessels was 0.518 ± 0.084 cm and 0.602 ± 0.099 cm at 7 days and 6 months after operation, respectively, in Group A. The velocity was 12.956 ± 2.402 cm/s and 13.084 ± 2.62 cm/s at 7 days and 6 months after operation, respectively. In Group B, 4 patients had a slightly larger diameter and the diameter slightly decreased in 1 patient at 6 months as compared to that at 7 days after operation. They were 0.79 cm, 0.51 cm, 0.62 cm, 0.53 cm, and 0.71 cm on 7 days and 0.82 cm, 0.59 cm, 0.60 cm, 0.58 cm, and 0.73 cm on 6 months.

**Fig. 4:**
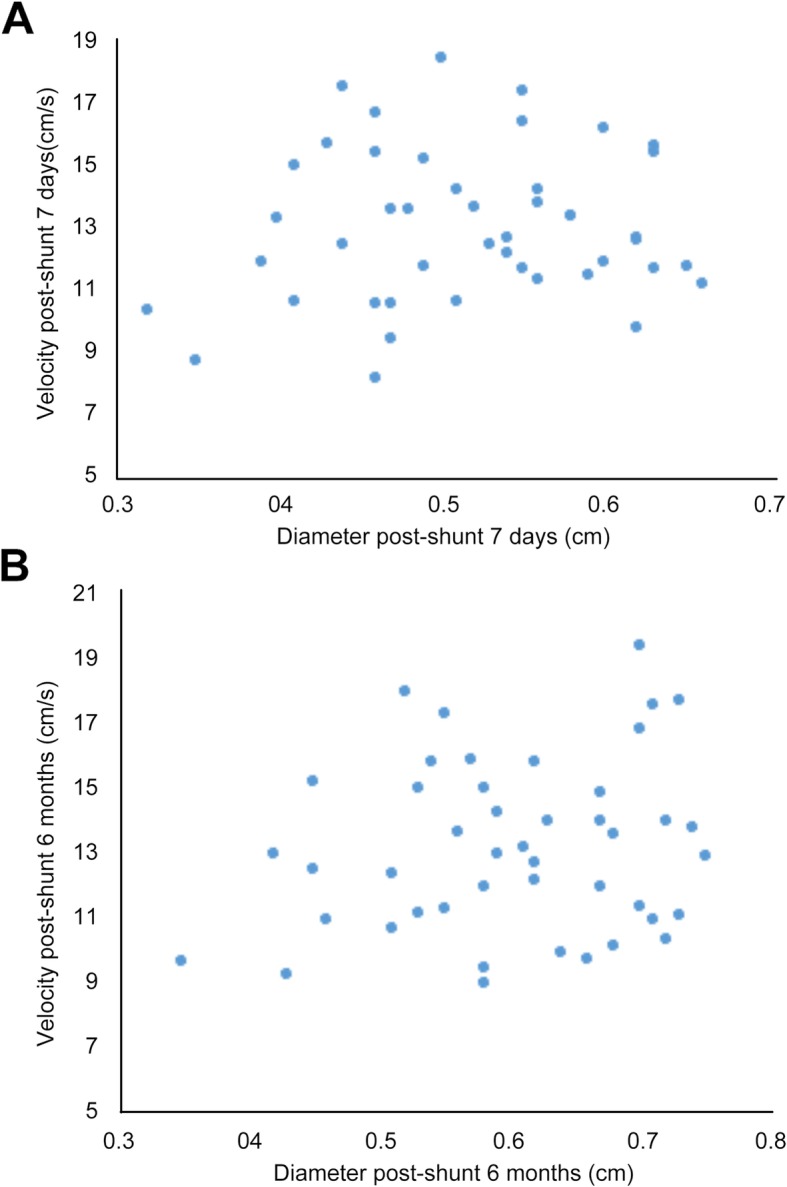
Distribution map of bypass diameter and flow velocity in Group A at 7 days (**a**) and 6 months (**b**) after Rex-bypass shunt operation

There was a significant difference in the diameter of bypass vessels between two time points after operation (*p* < 0.001). The diameter of bypass vessels at 6 months after operation was slightly larger than that at 7 days after operation. There was no significant difference in the blood flow velocity between two time points after operation (*P* = 0.507, Table 3). In order to analyze the relationship between bypass diameter and flow velocity in Group A, a distribution map was constructed (Figs. 4a, b). Pearson correlation analysis showed there was no significant correlation between bypass diameter and flow velocity (*r* = 0.102, *P* = 0.505).

**Table 3:** Changes of bypass diameter and flow velocity at 7 days and 6 months after operation in Group A

	7 days	6 months	*t*	*P*
Diameter (cm)	0.518 ± 0.084	0.602 ± 0.099	7.040	< 0.001
Velocity (cm/s)	12.956 ± 2.402	13.084 ± 2.62	0.669	0.507

## Discussion

CTPV is a collateral circulation to the hepatic vein formed after portal vein occlusion. Complete or partial portal vein obstruction may increase distal pressure and then collateral circulation. Extrahepatic portal vein obstruction is the most common cause of upper gastrointestinal bleeding in the children [14]. The etiology of extrahepatic portal vein obstruction in up to 50% of children remains unknown. The known causes include dehydration, intraabdominal infection, and hypercoagulable states [11, 15, 16].

Patients with CTPV may have portal hypertension symptoms such as upper gastrointestinal bleeding, hypersplenism, black stool, and ascites, while the pathological changes of the liver are not evident.

Rex shunt is an effective treatment for CTPV at an early stage in children. The surgery may reduce the portal pressure and the degree of esophagogastric varices, leading to the significant improvement of hypersplenism [3–5]. For patients with CTPV, the degree of esophagogastric varices is directly related to the upper gastrointestinal bleeding. The platelet count is an important parameter used for the assessment of hypersplenism. Color Doppler ultrasonography has shown excellent intrahepatic portal flow after Rex-bypass shunt [17]. In this study, the degree of esophagogastric varices on gastroscopy and the platelet count were determined. It has been shown the incidence of first postoperative rebleeding in children is higher within 6 months postoperatively than that at 6–12 months or 12 months after surgery [13]. Thus, in the present study, the degree of esophagogastric varices and platelet count were determined before and 6 months after Rex-bypass shunt in children with CTPV. In our study, 45 of 50 patients received color Doppler ultrasonography at 6 months after surgery and results showed good patency of the bypass vessels. The patency rate of bypass vessels was as high as 90%. The esophagogastric varices were significantly alleviated for patients with patent bypass vessels at 6 months after surgery (*p* < 0.05). This result was consistent with a previous study in which the success rate of Rex-bypass shunt was 91% and the gastrointestinal bleeding was significantly alleviated [18]. In addition, the platelet count significantly increased for patients with patent bypass vessels at 6 months after surgery (*p* < 0.001), which indicates that the hypersplenism is improved. These results indicate that color Doppler ultrasonography plays an important role in evaluating the effect of Rex-bypass shunt operation on the CTPV in children.

In five patients, blood flow signals and thrombosis were not observed in the bypass vessels at 6 months after Rex-bypass shunt operation, suggesting the bypass vessel obstruction. Although the number of patients is too small to statistically assess the bypass vessel obstruction, the degree of esophagogastric varices in these five patients remained unchanged after Rex-bypass shunt operation (Fig. 3, Table 2). This further confirms the value of color Doppler ultrasonography in assessing the patency of bypass vessels. In addition, four patients in Group B underwent splenorenal shunts subsequently.

There is evidence showing that the crossover bypass graft will dilate over time, which provides adequate blood outflow from the affected extremity in most patients suffering from post-thrombotic obstruction of the femoral and iliac veins [19]. A recent study revealed that the diameter of bypass vessels increased in 50% of CTPV children at 6 months after Rex-bypass shunt operation. The incidence of rebleeding and the degree of esophagogastric varices were significantly lowered after operation in patients with enlarged bypass vessels than in those without bypass vessel enlargement. This indicates that the improvement in patients with bypass vessel enlargement may be more evident [13]. In our study, the diameter of bypass vessels at 6 months after operation was slightly larger than that at 7 days after operation (39/45, *p* < 0.001). This was consistent with the above findings.

Blood velocity of the bypass vessel can be easily measured by color Doppler ultrasonography. Our results showed that the blood flow velocity of bypass vessels remained unchanged, although the bypass vessels dilated over time in most patients. This indicates that there was no linear relationship between the blood flow velocity and the diameter of the bypass vessel (*r* = 0.102, *P* = 0.505).

There are several limitations in this study. First, this is a retrospective study and only patients with ultrasound-confirmed CTPV were included. In a real situation, sometimes it is difficult to determine if a shunt is blocked. The bypass vessel obstruction was uncertain in six patients in the same study period we studied. These six patients underwent CTV subsequently. However, the results were still ambiguous. Thus, it is necessary to improve the accuracy of ultrasound diagnosis in the future. Second, only 26 patients received CTV, and CTV was unsuccessful in a few cases. Therefore, the diagnostic value between ultrasound and CTV could not be further evaluated in the study. Third, although all the cases in the study were examined by the same experienced clinician, the measurement errors of vessel diameter and blood flow velocity may not be completely avoided. In addition, long-term follow-up is needed to confirm the therapeutic efficacy of bypass shunt in these children.

## Conclusions

In conclusion, color Doppler ultrasonography can display the patency, diameter, and flow velocity of the bypass vessels and plays an important role in evaluating the effect of Rex-bypass shunt on the CTPV. As compared to CTV and portography, color Doppler ultrasonography is non-invasive, non-radioactive, and easy to perform. Thus, it can be used as a preferred tool for the assessment of CTPV after Rex-bypass shunt.

## Data Availability

The datasets used and/or analyzed during the current study are available from the corresponding author on reasonable request.

## Abbreviations

CTPV: Cavernous transformation of portal vein
